# Simultaneous siRNA-mediated knockdown of antiapoptotic BCL2, Bcl-xL, XIAP and survivin in bladder cancer cells

**DOI:** 10.3892/ijo.2012.1549

**Published:** 2012-07-06

**Authors:** DOREEN KUNZE, KAI KRAEMER, KATI ERDMANN, MICHAEL FROEHNER, MANFRED P. WIRTH, SUSANNE FUESSEL

**Affiliations:** Department of Urology, University Hospital Carl Gustav Carus, Technical University of Dresden, D-01307 Dresden, Germany

**Keywords:** apoptosis, combination therapy, RNA interference, small interfering RNA, siRNA transfection

## Abstract

Bladder cancer (BCa) represents the ninth most common malignancy worldwide. Despite intensive treatment with surgery and chemotherapy the prognosis for BCa patients particularly at advanced stages is poor. The ability to evade apoptosis is a hallmark of cancer cells. Since the antiapoptotic genes BCL2, Bcl-xL, XIAP and survivin are frequently upregulated in BCa tissues, their combined siRNA-mediated knockdown might be more potent in decreasing BCa growth than the single inhibition of one target. Against each target two siRNAs were selected that specifically reduced the mRNA and protein levels of their appropriate target in EJ28 and J82 BCa cells. Inhibition of survivin provoked the strongest antiproliferative effect of all single target treatments, for example cell counts decreased by 50%. Simultaneous targeting of all four antiapoptotic genes downregulated expression levels of all targets and mediated significant reductions in cell viability and cell counts as well as induction of apoptosis. In EJ28 cells, combined knockdown of BCL2, Bcl-xL, XIAP and survivin caused a 2.5-fold enhancement in apoptosis rate and reduced cellular viability by 40%. Therefore, simultaneous knockdown of antiapoptotic BCL2, Bcl-xL, XIAP and survivin may represent a promising treatment option for bladder cancer.

## Introduction

Bladder cancer (BCa) represents the ninth most common malignancy worldwide (1). For Europe, 139,500 new cases of BCa and 51,300 BCa-related deaths were estimated for the year 2008 (2). At the time of diagnosis 20–30% of patients present with muscle invasive disease and will be treated by radical cystectomy. Depending on the tumour stage, the 5-year survival rates range from 27 to 67% (3). For patients with metastatic BCa systemic chemotherapy is recommended. However, median survival of patients treated with gemcitabine and cisplatin is only 12.8 months (4). Altogether, despite intensive treatment with surgery and chemotherapy the prognosis for BCa patients particularly at advanced stages is poor. Therefore, the main goals of experimental BCa research are the improvement of existing therapies as well as the development of alternative therapeutic approaches.

One promising attempt is the knockdown of genes which play important roles in the survival or progression of cancer cells. Apoptosis (programmed cell death) is a highly conserved and strictly regulated biological process that is essential for the maintenance of normal tissue homeostasis as well as for the selective and non-inflammatory removal of infected or damaged cells (5). The ability to evade apoptosis is one of the hallmarks that characterise tumour cells (6). One mechanism by which cancer cells escape apoptosis is the overexpression of antiapoptotic genes, particularly of BCL2, Bcl-xL, XIAP and survivin.

BCL2 and Bcl-xL, two members of the BCL2 family, inhibit cell death by preventing the release of cytochrome *c* from the mitochondria and subsequent caspase activation (7). Activated caspases are the mediators of apoptosis which cleave proteins that are essential for cell function and stability (8). The detection of BCL2 protein in BCa tissue samples was generally associated with worse outcome (9–12). Bcl-xL positivity was found in 81% of BCa samples and correlated with high tumour stage and grade (13).

Survivin and XIAP are the two most important members of the inhibitor of apoptosis protein (IAP) family. XIAP executes its antiapoptotic function by direct inhibition of caspases (14). Sixty-one percent of nonmuscle-invasive BCa showed XIAP protein staining which was associated with a high risk of recurrence (15). Survivin is the fourth most common transcript found in human tumours (16). Survivin blocks cell death mainly by interactions with other proteins. For example, the survivin-XIAP complex enhances stability and activity of XIAP (17). Numerous studies documented the extraordinary importance of survivin for BCa diagnosis and prognosis as well as for the prediction of therapy response (reviewed in ref. 18). While survivin is absent in normal urothelium this IAP is found in 64–100% of BCa. Survivin expression is associated with high BCa stage and grade as well as with an elevated risk of recurrence (19–21).

The antiapoptotic factors BCL2, Bcl-xL, XIAP and survivin can protect BCa cells from natural cell death. Hence, the inhibition of these targets by small interfering RNAs (siRNAs) could cause the reactivation of apoptotic signalling and consequently a decrease in tumour growth. Small interfering RNAs are synthetic double-stranded ribonucleic acids that mediate specific gene silencing by inducing RNA interference (22). Thereby, siRNAs are bound into the RNA-induced silencing complex (RISC) which mediates the cleavage of the target mRNA by its intrinsic endonuclease function (23). Since cancer cells may bypass the inhibition of one target, e.g. a reduction in BCL2 protein content can be compensated by the induction of Bcl-xL (24), the combined knockdown of antiapoptotic genes may be more suitable for BCa therapy. Therefore, we analysed effects of single and combined siRNA-mediated knockdown of BCL2, Bcl-xL, XIAP and survivin on human BCa cell lines.

## Materials and methods

### Cell culture, siRNAs and transfection

The human BCa cell lines EJ28 (University of Frankfurt, Frankfurt, Germany), J82 and 5637 (ATCC, Manassas, VA, USA) were cultured under standard conditions (37°C, humidified atmosphere containing 5% CO_2_) without antibiotics. For EJ28 and J82, DMEM (4.5 g/l glucose) containing 10% fetal calf serum (FCS), 1% MEM non-essential amino acids and 1% HEPES (all from Invitrogen, Karlsruhe, Germany) was used. 5637 cells were cultured in RPMI-1640 (Invitrogen) including 10% FCS and 1% MEM non-essential amino acids.

The target-directed siRNAs (abbreviations are shown in Table I) and the negative control siRNA ‘ns-si’ (reference: SR-CL000-005), which was used for normalisation, were synthesised by Eurogentec (Seraing, Belgium). After seeding and adherence for 24 or 72 h, cells were washed with PBS and transfected with the siRNAs for 4 h in serum-free OptiMEM (Invitrogen) using DOTAP liposomal transfection reagent (ratio 1:30, w/w) according to the manufacturer’s instructions (Roche, Mannheim, Germany). Unless otherwise stated, the siRNAs were transfected either separately with 40 nM of one construct (single target treatments) or with combinations of four (M4-1 and M4-2, 10 nM per siRNA) or all eight target-directed siRNAs (M8, 5 nM per siRNA). In the M4 combination treatments the siRNAs B2-1, BX-1, X-1 as well as S-1 (= M4-1) or B2-2, BX-2, X-2 as well as S-2 (= M4-2) were incubated simultaneously. After 4 h, transfection medium was replaced by fresh culture medium and cells were incubated for 24–96 h. For further analyses cells were harvested by trypsin treatment (0.05% trypsin/0.02% EDTA, 5 min, 37°C). Detached and adherent cells were pooled and analysed together.

### Viability assay, apoptosis detection and cell cycle analysis

Using the cell proliferation reagent WST-1 (Roche) cellular viability was examined in quadruplicates 96 h after transfection according to the manufacturer’s instructions. Apoptosis was assessed by Annexin V-FITC/propidium iodide (PI) staining (Annexin V-FITC Apoptosis Detection Kit I, BD Biosciences, Heidelberg, Germany) 48 h after transfection according to the manufacturer’s instructions by flow cytometry (FACScan, BD Biosciences). Percentage of early (Annexin V-FITC positive, PI negative) and late (Annexin V-FITC positive, PI positive) apoptotic cells was determined by quadrant analysis of Annexin V-FITC/PI plots using WinMDI2.8 software (http://facs.scripps.edu/software.html). Cell cycle distribution was assessed by propidium iodide staining (CycleTest Plus DNA Reagent Kit, BD Biosciences) 48 h after transfection according to the manufacturer’s instructions by flow cytometry (FACScan, BD Biosciences).

### RNA isolation, cDNA synthesis and quantitative PCR

Total-RNA was isolated according to the manufacturer’s instructions (InviTrap Spin Cell RNA Mini Kit; Invitek, Berlin, Germany) and reverse transcribed into cDNA (SuperScript II Reverse Transcriptase; Invitrogen). Transcript amounts of the targets and the reference gene TBP (TATA box binding protein) were determined by quantitative real-time PCR (qPCR) using the primers, probes and kits listed in Table II.

### Western blot analysis

Cells (5x10^4^ per sample) were lysed in 20 µl loading buffer (20% glycerol, 2% SDS, 125 mM Tris pH 6.8, 5% β-mercaptoethanol, bromophenol blue), incubated at 95°C for 5 min and separated on 8–16% Precise Protein Gels (Fisher Scientific, Schwerte, Germany). Proteins were transferred onto PVDF membranes (GE Healthcare, Freiburg, Germany) which were incubated with primary antibodies against BCL2 (1:200; clone 124; Dako, Glostrup, Denmark), Bcl-xL (1:100; clone 2H12; QED Bioscience Inc., San Diego, CA, USA), XIAP (1:250; clone 28; BD Biosciences) or survivin (1:1,000; NB500-201; Novus Biologicals, Littleton, CO, USA). β-actin detected by a monoclonal anti-β-actin antibody (1:20,000; Sigma-Aldrich, St. Louis, MO, USA) served as a loading control. The secondary polyclonal rabbit anti-mouse immunoglobulin HRP-linked antibody (1:1,000; Dako; for β-actin, BCL2, Bcl-xL and XIAP) or the polyclonal swine anti-rabbit immunoglobulin HRP-linked antibody (1:1,000, Dako, for survivin), respectively, as well as the Enhanced chemiluminescence Kit (GE Healthcare) were used for visualization.

## Results

### BCL2, Bcl-xL, XIAP and survivin expression in bladder cancer cell lines

Quantitative PCR analysis was used to determine expression levels of the four selected antiapoptotic genes in EJ28, J82 and 5637 BCa cells. As shown in Table III, BCL2, Bcl-xL, XIAP and survivin are expressed simultaneously in all BCa cell lines. Since EJ28 and J82 cells express all targets at high levels these cell lines were chosen for target inhibition studies.

### Optimising siRNA transfection for combined knockdown of four target genes

**Figure 1:**
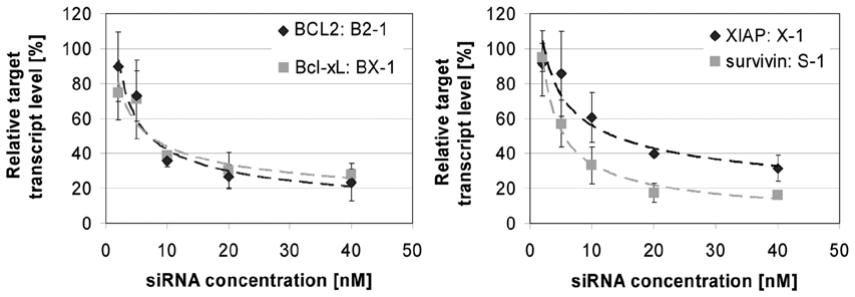
Reduction of BCL2, Bcl-xL, XIAP and survivin mRNA levels dependent on the concentration of the siRNA 24 h after transfection with the appropriate target-specific siRNA in EJ28 BCa cells. Cells were transfected with 2, 5, 10, 20 or 40 nM target-specific or control siRNA. Values are normalised to the reference gene TBP and are shown relative to the respective control siRNA treatment (100%). Values represent averages of two independent experiments with their mean deviation.

To achieve strong target inhibition and to avoid undesirable side effects, utilisation of highly effective siRNAs at low concentrations is recommendable. Therefore, target knockdown depending on the siRNA concentration was examined using the constructs B2-1, BX-1, X-1 and S-1 in EJ28 cells. Already by using 10 nM of the siRNAs a marked reduction of the target mRNA levels by 40–67% was achieved 24 h after treatment (Fig. 1). A quadruplication of the applied siRNA concentration increased the target inhibition rate to 69–84% (Fig. 1). To ensure comparability among single target and target combination treatments, an equal total amount of siRNAs as well as an equal amount of DOTAP transfection reagent was used. Therefore, a final concentration of 40 nM siRNA was selected, whereby 40 nM siRNA were used in the single target treatments and 10 nM (M4-1, M4-2) or 5 nM (M8) per siRNA in the combinations. Thus, a marked target inhibition in all treatments is ensured while side effects are maintained at the same level by constant amounts of DOTAP.

### Molecular effects of single and combined siRNA-mediated target inhibition

**Figure 2:**
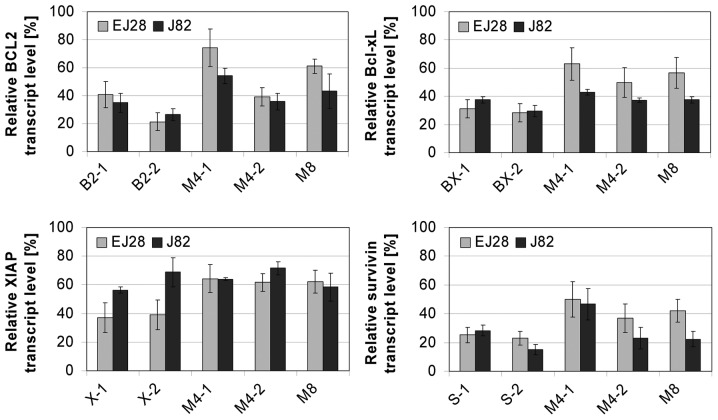
Relative target mRNA expression levels of EJ28 and J82 bladder cancer cells 48 h after transfection with a total of 40 nM siRNA. Expression values are normalised to the reference gene TBP and are shown relative to the control siRNA (100%). Values represent averages of two independent experiments with their mean deviation.

**Figure 3:**
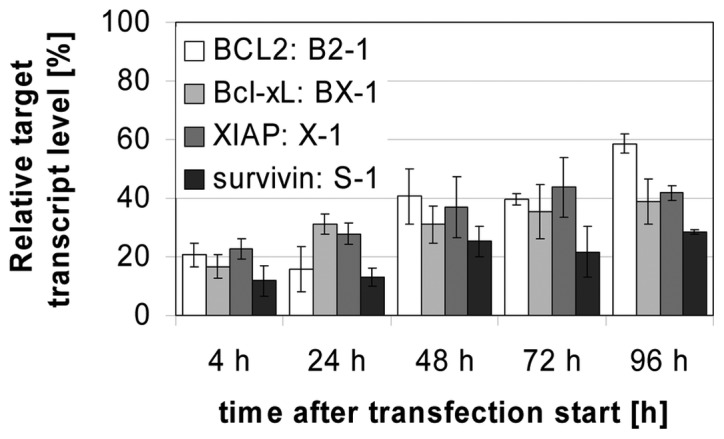
Time-dependent reduction of BCL2, Bcl-xL, XIAP and survivin mRNA levels after transfection with 40 nM of the appropriate target-specific siRNA in EJ28 BCa cells. Values are normalised to the reference gene TBP and are shown relative to the control siRNA (100%). Values represent averages of two independent experiments with their mean deviation.

**Figure 4:**
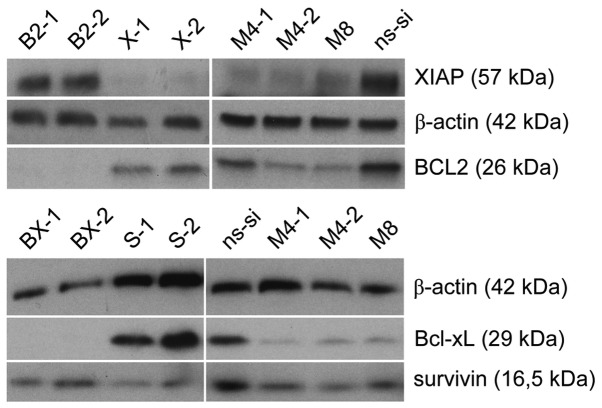
Detection of BCL2, Bcl-xL, XIAP and survivin protein content by western blotting 48 h after transfection with a total of 40 nM siRNA in EJ28 bladder cancer cells. Beta-actin was used for loading control.

The selected target-specific siRNAs (Table I) potently decreased the mRNA expression levels of their appropriate target in both BCa cell lines 48 h after transfection (Fig. 2). In EJ28 cells, BCL2 was reduced down to 21%, Bcl-xL down to 28%, XIAP down to 37% and survivin down to 23% at best. Even 96 h after transfection a target inhibition down to 28% (survivin) and to 59% (BCL2) was measured (Fig. 3). The simultaneous inhibition of all four antiapoptotic genes resulted in mRNA downregulation of all targets. Using a combination of all eight siRNAs, BCL2 was reduced down to 61 and 43%, Bcl-xL down to 57 and 38%, XIAP down to 62 and 59%, and survivin down to 42 and 22% in EJ28 and J82 BCa cells, respectively (Fig. 2). Western blot analysis showed specific protein reduction 48 h after siRNA transfection in single target and target combination treatments in both BCa cell lines (Fig. 4, representative western blots are shown for EJ28 cells, similar results were obtained with J82 cells).

### Cellular effects of single and combined siRNA-mediated target inhibition

**Figure 5:**
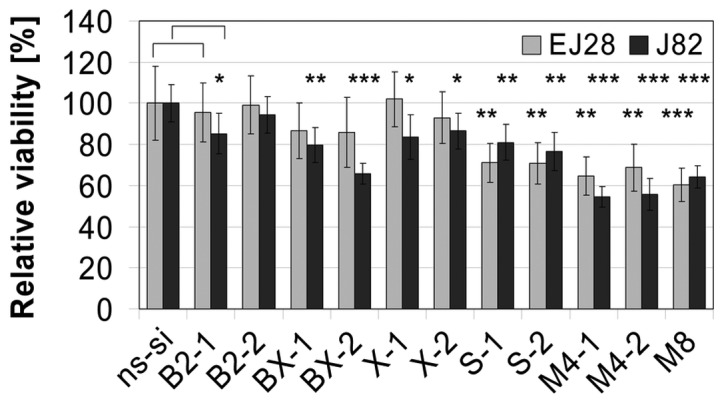
Viability of EJ28 and J82 bladder cancer cells 96 h after transfection with a total of 40 nM siRNA. Values shown are relative to the control siRNA ‘ns-si’ (100%) and are averages of a fourfold determination. Error bars represent the 95% confidence interval. An unpaired Student’s t-test was used to compare the differences in cell viability between target-directed-siRNA and ns-si treated cells (^*^p≤0.05, ^**^p≤0.01, ^***^p≤0.001).

**Figure 6:**
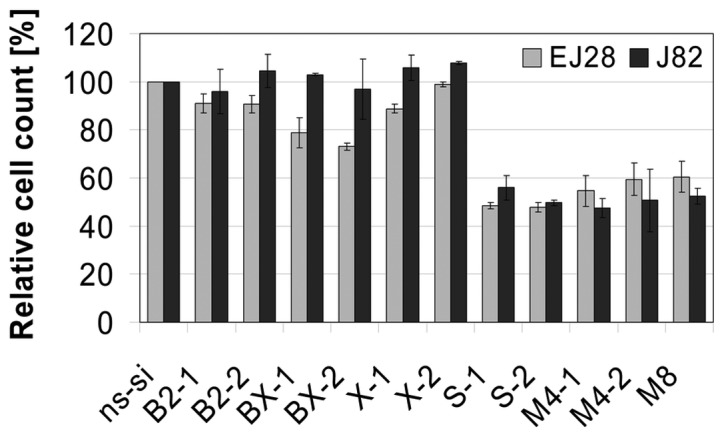
Cell count of EJ28 and J82 bladder cancer cells 48 h after transfection with a total of 40 nM siRNA. Values shown are relative to the control siRNA ‘ns-si’ (100%) and are averages of two independent experiments. Error bars represent the mean deviation.

Inhibition of BCL2 and XIAP did not or only marginally affect the growth of the BCa cell lines EJ28 and J82 (Figs. 5 and 6). Knockdown of Bcl-xL reduced BCa cell viability by 13–34% (Fig. 5). Strongest decrease in cell viability as well as a profound reduction in cell counts were observed after siRNA-mediated inhibition of survivin (Figs. 5 and 6). Antiproliferative effects of the siRNA combination treatments M4-1, M4-2 and M8 with one or two siRNAs per target were comparable (Figs. 5 and 6). On average, simultaneous knockdown of BCL2, Bcl-xL, XIAP and survivin inhibited BCa cell viability by 39% and BCa cell counts by 46%.

**Figure 7:**
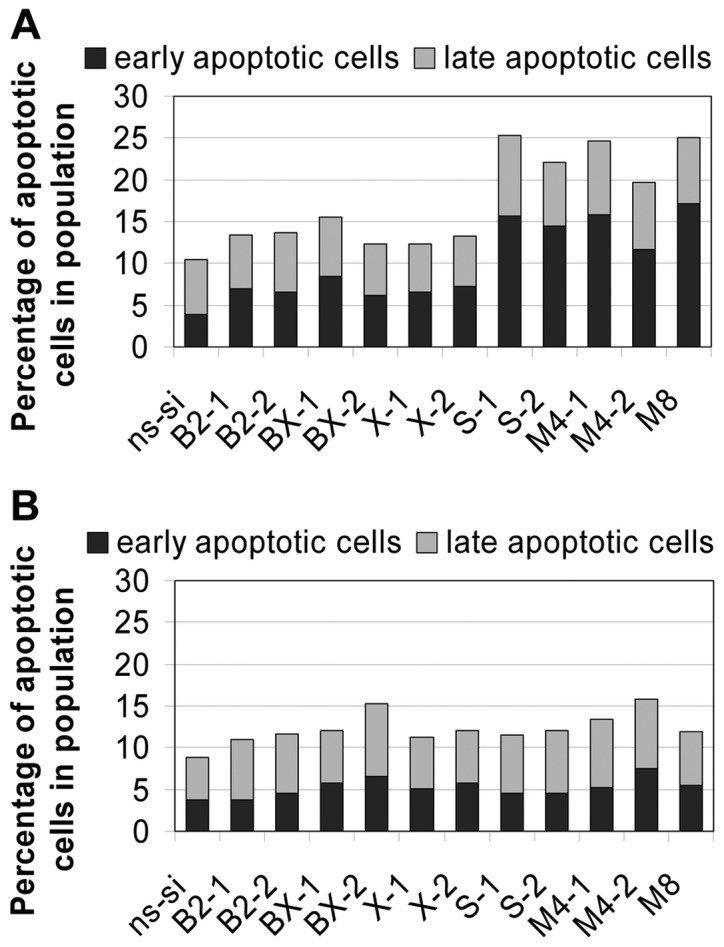
Percentage of early and late apoptotic cells 48 h after treatment of (A) EJ28 and (B) J82 cells with a total of 40 nM siRNAs. Values shown are representatives of two independent experiments.

In EJ28 cells single knockdown of survivin as well as simultaneous inhibition of all four antiapoptotic genes caused a 1.9 to 2.5-fold enhancement in apoptosis rate (Fig. 7A). For example, percentage of apoptotic cells in population increased from 10% in the ns-si control to 25% after treatment with the anti-survivin siRNA S-1. In J82 cells only a marginal enhancement of apoptosis by factor 1.4 on average was seen (Fig. 7B). No changes in cell cycle distribution were found in EJ28 and J82 cells after inhibition of BCL2, Bcl-xL or XIAP whereas knockdown of survivin caused polyploidy in both BCa cell lines (data not shown). For example, 10% of the EJ28 cells showed DNA content of 8N after treatment with S-1 or S-2 compared to 1% in the ns-si control. In the combination treatments M4-1, M4-2 and M8 similar changes were found but effects were less prominent, e.g. 48 h after transfection with M4-2, 2 and 4% of EJ28 and J82 cells, respectively, showed DNA content of 8N.

## Discussion

Deregulation of apoptosis is a key factor in tumourigenesis (6). The members of the BCL2 and IAP families are of particular importance for the regulation of apoptotic signalling (25). BCL2, Bcl-xL, XIAP and survivin are the most important antiapoptotic members of these two families and are frequently upregulated in human tumours including BCa (15,26,27). Therefore, these genes represent interesting candidates for a target-directed molecular-based antitumour therapy. Since the knockdown of a single antiapoptotic target might be bypassed by the upregulation of other prosurvival genes the simultaneous inhibition of BCL2, Bcl-xL, XIAP and survivin could be more potent in decreasing BCa cell proliferation.

Using 40 nM of new siRNAs with optimised design, comparable mRNA inhibition rates were obtained in the single target treatments as with much higher concentrations of different siRNAs targeted at BCL2, Bcl-xL, XIAP or survivin in previous studies. Only 40 nM of B2-1, BX-1 and X-1 reduced mRNA levels of their appropriate target down to 59, 39 and 42% in EJ28 cells 96 h after transfection (Fig. 3) whereas 200 nM of the previously used siRNAs (28) decreased mRNA levels down to 59, 37 and 46%, respectively. Reduction in survivin expression of about 70 and 50% was shown in EJ28 and J82 BCa cells, respectively, 48 h after transfection with 250 nM siRNA (29). In the present study, survivin mRNA was decreased with 40 nM of the new siRNAs in EJ28 and J82 cells by 76 and 79% on average, respectively (Fig. 2). Even 96 h after treatment target mRNA levels were downregulated by up to 72% (Fig. 3). These facts verify that the novel siRNAs induced an effective and long-lasting inhibition of their target expression. In addition, the risk of undesired side effects is minimised due to the significantly lower siRNA concentrations applied.

The simultaneous transfection of various siRNAs might induce a competition between the constructs as to their incorporation into RISC (30). Therefore, the effectiveness of individual siRNAs might be limited in combination treatments. With the siRNAs used in this study no competition between the constructs regarding their incorporation into RISC is assumed. The slightly decreased mRNA inhibition rate in the combination treatments (M4-1, M4-2, M-8) compared to the single target treatments seemed to be mediated basically by the different siRNA concentrations with 40 nM per siRNA in the single target treatments and 10 nM siRNA per target in the combinations treatments (Fig. 2). Comparably, Yang *et al* demonstrated effective protein knockdown of the three IAPs livin, XIAP and survivin with a siRNA combination comprising of 10 nM siRNA per target. The protein reduction in the combination treatment was only slightly decreased in comparison to the single target treatments with 30 nM siRNA (31).

All siRNAs used in the present study, either separately or combined, sufficiently decreased the mRNA and protein levels of their targets (Figs. 2 and 4). Of the single target treatments, the inhibition of survivin caused strongest antiproliferative effects on EJ28 and J82 BCa cells, namely considerable reductions in cell viability and cell counts (Figs. 5 and 6). This is mediated by apoptosis induction and the formation of polyploid cells. Survivin knockdown induces polyploidy because survivin is, besides its function as inhibitor of apoptosis, an integral part of the chromosomal passenger complex, thereby regulating chromosome segregation and cytokinesis (32). In agreement with the results of this study, Ning *et al* and Takizawa *et al* showed apoptosis induction and an arrest in G2/M cell cycle phase after treatment of BCa cells with siRNAs targeted at survivin (29,33).

Despite marked decreases in target protein contents (Fig. 4), BCL2 and XIAP single knockdown had no or only marginal impact on BCa cell growth (Figs. 5 and 6). Using other siRNA sequences previous studies showed a reduction in EJ28 cell counts of about 43% after transfection of 200 nM siRNA targeting BCL2 or XIAP (28). Since target mRNA inhibition rates were comparable to the present study, differences might be due to off-target effects mediated by higher siRNA concentrations. Similarly to the present results, Sensintaffar *et al* showed that siRNA-induced XIAP knockdown did not affect T24 BCa cell viability 72 and 96 h after treatment (34), neither did BCL2 inhibition in prostate cancer cell lines induce phenotypic changes (35). Possibly, BCL2 and XIAP are of minor importance in several monolayer cell cultures due to their continuous supply with oxygen and nutrients. In contrast, cancer cell aggregates like tumour spheroids that represent better models for tumour structure contain nutrient-deficient and hypoxic microenvironments which might show differing gene expression profiles. Indeed, increased BCL2 and XIAP protein contents were found in tumour spheroids of lung and breast cancer cells, respectively, in comparison to the corresponding monolayer cell cultures (36,37). Moreover, inhibition of BCL2 and XIAP might not induce direct antiproliferative effects in cancer cells but rather a sensitisation to exogenous apoptosis stimuli similar to chemotherapy or radiation.

Bcl-xL reduction in BCa cells induced moderate antiproliferative effects. For example in EJ28 cells, viability decreased by 14% and apoptosis rate increased on average by 34% relative to negative control siRNA treated cells (Figs. 5 and 7). In the same cell line 200 nM of another Bcl-xL targeting siRNA induced apoptosis and sensitised cells towards a subsequent chemotherapy with mitomycin C (28). Similarly, in prostate and ovarian cancer cells siRNA-mediated Bcl-xL knockdown inhibited tumour cell proliferation and sensitised cells towards cisplatin and tumour necrosis factor-related apoptosis-inducing ligand (TRAIL), respectively (38,39).

Combined inhibition of BCL2, Bcl-xL, XIAP and survivin was carried out by simultaneous transfection of one (M4-1, M4-2) or two (M8) siRNAs per target. All three combination treatments induced comparable cellular effects (Figs. 5–7). After simultaneous inhibition of all four targets, effective reductions in cell viability and cell counts as well as induction of apoptosis were seen. These effects were as strong as after survivin knockdown, which was the most efficient single target treatment. Percentage of polyploid BCa cells increased in the combination treatments but was lower than after survivin single knockdown. Because survivin is the only target which additionally functions in cytokinesis, the formation of polyploid cells should represent the consequence of survivin knockdown. Since downregulation of survivin in the single target treatments with 40 nM siRNA is slightly stronger than after simultaneous inhibition of BCL2, Bcl-xL, XIAP and survivin with 10 nM siRNA per target (Fig. 2), the proportion of polyploid cells in the combination treatments might be lower because of the higher amount survivin remaining. That the degree of BCa growth reductions in the M4 and M8 treatments is not different from the value after single survivin inhibition should be the consequence of a synergistic action of the simultaneous knockdown of multiple antiapoptotic genes, presumably survivin and Bcl-xL.

Further studies showed the potential of the simultaneous knockdown of multiple antiapoptotic genes. The combined targeting of the three IAPs c-IAP1, c-IAP2 and XIAP in prostate cancer cells decreased proliferation and sensitised cells to TRAIL treatment (40). In pancreatic cancer cells, the simultaneous inhibition of BCL2, XIAP and survivin mediated induction of apoptosis (41). Moreover, Yang *et al* demonstrated that the combined knockdown of livin, XIAP and survivin in T24 BCa cells reduced cell proliferation and induced apoptosis (31). These studies as well as the present report prove that the simultaneous inhibition of multiple antiapoptotic genes might be a promising treatment option for cancer.

## Figures and Tables

**Table I:** Designations and target sequences of the siRNAs.

Target gene	siRNA name	siRNA target sequence
BCL2	B2-1	CGGUGGUGGAGGAGCUCUU
BCL2	B2-2	GCAUGCGGCCUCUGUUUGA
Bcl-xL	BX-1	GGGACAGCAUAUCAGAGCU
Bcl-xL	BX-2	CAGCUGGAGUCAGUUUAGU
XIAP	X-1	CGAGCAGGGUUUCUUUAUA
XIAP	X-2	CUGGGCAGGUUGUAGAUAU
Survivin	S-1	GAAGCAGUUUGAAGAAUUA
Survivin	S-2	CCAACAAUAAGAAGAAAGA

All siRNAs have 3′-dT overhangs.

**Table II:** Sequences of primers and probes and the kits used for quantitative PCR.

Target	Sequence 5′→3′
BCL2^a^	Target-specific Real-Time Reagent Mix (AJ Roboscreen, Leipzig, Germany) containing the appropriate primers and probes
Bcl-xL^a^	
Survivin^b^	Primers: for: GAACTGGCCCTTCTTGGAG, rev: AAGTCTGGCTCGTTCTCAGTG
	Probe: Universal ProbeLibrary Probe no. 86 (Roche, Germany, cat. no. 04689119001)
TBP^a^	Primers: for: GAATATAATCCCAAGCGGTTTG, rev: ACTTCACATCACAGCTCCCC
	Probes: TTTCCCAGAACTGAAAATCAGTGCC-FL, LC-TGGTTCGTGGCTCTCTTATCCTCATG-PH
XIAP^a^	Primers: for: GTGATAAAGTAAAGTGCTTTCACTGT, rev: GTAGTTCTTACCAGACACTCCTCAA
	Probes: GTGAAGACCCTTGGGAACAACAT-FL, LC-CTAAATGGTATCCAGGGTGCAAATATCTG-PH

a LightCycler FastStart DNA Master Hybridization Probes (Roche);

b LightCycler TaqMan Master (Roche); FL, fluorescence dye fluorescein; LC, fluorescence dye LC Red640; PH, phosphorylated 3′-end.

**Table III:** Target mRNA expression levels in EJ28, J82 and 5637 bladder cancer cell lines.

Cell line	BCL2/TBP	Bcl-xL/TBP	XIAP/TBP	Survivin/TBP
EJ28	0.283	27.8	3.25	4.35
J82	0.084	24.0	2.00	2.77
5637	0.058	7.0	1.92	1.10

Values are normalised to the reference gene TBP.
